# In Vitro and In Vivo Short-Term Pulmonary Toxicity of Differently Sized Colloidal Amorphous SiO_2_

**DOI:** 10.3390/nano8030160

**Published:** 2018-03-13

**Authors:** Martin Wiemann, Ursula G. Sauer, Antje Vennemann, Sandra Bäcker, Johannes-Georg Keller, Lan Ma-Hock, Wendel Wohlleben, Robert Landsiedel

**Affiliations:** 1 IBR R&D gGmbH Institute for Lung Health, Mendelstr. 11, 48149 Münster, Germany; vennemann@ibe-ms.de; 2 Scientific Consultancy—Animal Welfare, 85579 Neubiberg, Germany; ursula.sauer@sauerug.de; 3 BASF SE, Human Biomonitoring and Industrial Hygiene, 67056 Ludwigshafen, Germany; sandra.baecker@basf.com; 4 BASF SE, Advanced Materials Research, 67056 Ludwigshafen, Germany; johannes-georg.keller@basf.com (J.-G.K.); wendel.wohlleben@basf.com (W.W.); 5 BASF SE, Experimental Toxicology and Ecology, 67056 Ludwigshafen, Germany; lan.ma-hock@basf.com (L.M.-H.); robert.landsiedel@basf.com (R.L.)

**Keywords:** alveolar macrophage, short-term inhalation study (STIS), intratracheal instillation, in vitro cytotoxicity, 3R method, dosimetry, TNFα, nanomaterial grouping, regulatory hazard assessment

## Abstract

In vitro prediction of inflammatory lung effects of well-dispersed nanomaterials is challenging. Here, the in vitro effects of four colloidal amorphous SiO_2_ nanomaterials that differed only by their primary particle size (9, 15, 30, and 55 nm) were analyzed using the rat NR8383 alveolar macrophage (AM) assay. Data were compared to effects of single doses of 15 nm and 55 nm SiO_2_ intratracheally instilled in rat lungs. In vitro, all four elicited the release of concentration-dependent lactate dehydrogenase, β-glucuronidase, and tumor necrosis factor alpha, and the two smaller materials also released H_2_O_2_. All effects were size-dependent. Since the colloidal SiO_2_ remained well-dispersed in serum-free in vitro conditions, effective particle concentrations reaching the cells were estimated using different models. Evaluating the effective concentration–based in vitro effects using the Decision-making framework for the grouping and testing of nanomaterials, all four nanomaterials were assigned as “active.” This assignment and the size dependency of effects were consistent with the outcomes of intratracheal instillation studies and available short-term rat inhalation data for 15 nm SiO_2_. The study confirms the applicability of the NR8383 AM assay to assessing colloidal SiO_2_ but underlines the need to estimate and consider the effective concentration of such well-dispersed test materials.

## 1. Introduction

Engineered nanomaterials encompass a large variety of inorganic and organic chemicals. An abundance of nanoforms that differ, such as in primary particle size (PPS), shape, or surface function, can be produced for any given nanomaterial [1,2]. While an increasing number of scientific publications address safety assessments of engineered nanomaterials [3,4,5,6,7], it is widely acknowledged that toxicity testing to meet full regulatory information requirements, e.g., in accordance with Regulation (EC) No 1907/2006 on the Registration, Evaluation, Authorisation, and Restriction of Chemicals (REACH [8]), for every variant of a given nanomaterial would lead to an insurmountable amount of testing [9]. This would further stand in contradiction to the ethical and legal requirement to replace, reduce, and refine animal testing (3Rs principle) [10,11].

For many nanomaterials, inhalation is the predominant route of exposure [5,6,12]. A rat short-term inhalation study (STIS) is available that allows reducing and refining the use of animals as compared to the traditional Organisation for Economic Cooperation and Development (OECD) test guideline (TG) 412, Sub-acute inhalation toxicity: 28-day study [12,13,14,15]. By comparison, standardized in vitro assays to assess the cellular effects of nanomaterials continue to be unavailable [6,7,16,17,18]. Only a few of the numerous published in vitro studies investigating the cellular effects of nanomaterials were aimed at predicting in vivo toxicity potential [1,6,7,19,20,21,22].

Recently, a rat NR8383 alveolar macrophage (AM) assay has proven useful in predicting the short-term inhalation toxicity potential of nanomaterials and non-nanosized materials [22]. AMs serve as the first line of host defense against inhaled particles [23,24,25,26]. The NR8383 AM assay was founded on an assay originally developed for primary AMs by Rehn et al. [26,27,28]. In Wiemann et al. [22], the concept of the original assay was adapted to cultured rat NR8383 AMs that maintain their typical AM-like size and appearance, along with phagocytic and many immunological properties [29,30,31].

Due to its high predictivity of the short-term inhalation toxicity of nanomaterials, the NR8383 AM assay is the recommended in vitro assay in the Decision-making framework for the grouping and testing of nanomaterials (DF4nanoGrouping) [32,33]. The grouping of nanomaterials (or other substances) is widely recognized as a means to streamline regulatory testing needs [1,4,34,35,36,37,38], and the DF4nanoGrouping provides a comprehensive framework to assign nanomaterials to one of four main groups, termed MG1 to MG4, as explained in Box 1 [32,33].

Box 1 **The DF4nanoGrouping [32,33].**

In Tier 1 of the DF4nanoGrouping, three intrinsic material properties are evaluated, i.e., water solubility, particle morphology and chemical composition.

In Tier 2, three system-dependent properties are evaluated, i.e., dissolution in biological media, surface reactivity, and particle dispersibility, as well as in vitro cellular effects for which the NR8383 AM assay is the recommended test method.

Together, these grouping criteria allow identifying all nanomaterials as either (MG1) soluble, (MG2) biopersistent high aspect ratio, (MG3) passive, or (MG4) active nanomaterials.

If necessary, the outcome of the non-animal Tiers 1 and 2 is verified in Tier 3 using in vivo STIS data that also serve to sub-group the (MG4) active nanomaterials to specify the further testing needs.

Extensive case studies that also included different amorphous SiO_2_ nanomaterials confirmed the usefulness of the DF4nanoGrouping for hazard and risk assessment [33]. Amorphous SiO_2_ nanomaterials are widely used, e.g., in cement, paint, cosmetics, and food, and are produced using a variety of methods [3,39,40]. The outcome of the DF4nanoGrouping case studies highlighted the need to further investigate how different modifications of the same amorphous SiO_2_ can affect their intrinsic material properties, system-dependent properties, and toxicity potential [33].

Against this background, the present study aimed to evaluate the in vitro cellular effects and in vivo short-term pulmonary toxicity of colloidal amorphous SiO_2_ nanomaterials that differed only by PPS, and hence surface area: Levasil^®^ 300/30% (9 nm SiO_2_), Levasil^®^ 200/40% (15 nm SiO_2_), Levasil^®^ 100/45% (30 nm SiO_2_), and Levasil^®^ 50/50% (55 nm SiO_2_). These stable colloidal dispersions are synthesized by means of a growth process from an aqueous solution with dissociated molecular silicic acid [41]. Of note, whereas the Levasil^®^ numberings relate to the respective materials’ specific surface areas, the terms 9 nm SiO_2_, 15 nm SiO_2_, etc., reflect their PPS. Larger Levasil^®^ numberings (and thus surface areas) correspond to smaller PPSs.

By selecting four differently sized but otherwise identical test materials, the present study allowed for assessment of how the PPS of colloidal SiO_2_ affects its intrinsic material properties, system-dependent properties (dispersibility, dissolution rate, and surface reactivity, with a focus on behavior under cell culture conditions), and in vitro and in vivo pulmonary toxicity. Previous studies have attempted to correlate nanoparticle size or specific nanomaterial surface properties with toxic effects [40,42,43,44,45,46,47,48,49,50,51]. Specific correlations could not be shown consistently for different types of test materials with particle sizes of 1–100 nm, the range used in regulatory nanomaterial definitions [52,53,54]. In the DF4nanoGrouping, particle size has been identified as a supplementary grouping criterion [32,33]. In vivo, the pulmonary toxicity of nanomaterials appears to be mainly explained by their chemical composition and/or surface reactivity [5,55].

For in vitro assessment of 9 nm SiO_2_, 15 nm SiO_2_, 30 nm SiO_2_, and 55 nm SiO_2_, four cellular effects were measured in the NR8383 AM assay: extracellular release of (1) H_2_O_2_, reflecting the synthesis of reactive oxygen species (ROS); (2) lactate dehydrogenase (LDH), reflecting enzyme leakage from the cytosol; (3) β-glucuronidase (GLU), reflecting activation and/or membrane damage of the phagolysosomal compartment; and (4) biologically active tumor necrosis factor alpha (TNFα) as a major proinflammatory cytokine [22]. In accordance with the NR8383 AM assay protocol [22], the cellular particle burden was estimated, because colloidal SiO_2_ nanomaterials show minimal gravitational settling [22]. Therefore, the in vitro test results obtained in the present study are expressed as nominal (total applied) concentration. In addition, the effective concentration, i.e., the proportion of nanoparticles in the homogeneous suspension that ended up in the bottom area of the cell culture vessel where the nanoparticles might reach the cells, was calculated [56,57,58].

For in vitro–in vivo comparison of test results, 15 nm SiO_2_ and 55 nm SiO_2_ were further evaluated in an in vivo rat intratracheal instillation study [59]. Intratracheal instillation studies are considered useful in vivo screening tests, since the resulting lung burden is equal to the bolus dose of the test material. Finally, the outcomes of the NR8383 AM assay and the rat intratracheal instillation study were compared to published STIS data for 15 nm SiO_2_ [15].

## 2. Results

### 2.1. Test Materials and Test Material Characterization

Table 1 provides an overview of the intrinsic material properties and system-dependent properties of 9 nm SiO_2_, 15 nm SiO_2_, 30 nm SiO_2_, and 55 nm SiO_2_. Compared to the manufacturer’s specification of PPS, an increase in particle size of only 40–60% was observed when the test materials were suspended in protein-free F-12K medium (the main cell culture medium used in the present study), Krebs-Ringer phosphate glucose (KRPG) buffer (used to assess in vitro H_2_O_2_ synthesis and release), or 0.9% NaCl solution (used to prepare the test materials in the intratracheal instillation study). The dispersed sizes of the respective test materials were nearly identical between these three protein-free media, indicating strong stabilization of all colloidal amorphous SiO_2_ by negative charge. Only in serum containing Dulbecco’s Modified Eagle Medium (DMEM) supplemented with 10% fetal calf serum (FCS), which is not used in the NR8383 AM assay, were strong interaction and agglomeration recorded.

Water solubility of the test materials, determined by inductively coupled plasma–mass spectrometry (ICP-MS) after force filtration, as described in OECD [60], decreased with increasing PPS (from 48 mg/L for 9 nm SiO_2_ to 7 mg/L for 55 nm SiO_2_; Table 1). In flow-through testing of the dissolution rate of the test materials in phagolysosomal simulant fluid (pH 4.5), all four colloidal SiO_2_ exhibited minimal dissolution. Interestingly, the concentration of ionic species increased nonlinearly with increasing surface area, suggesting a transformation of the particulate species, such as by gel formation [61].

All four test materials exhibited a similar strongly negative charge (Table 1): 9 nm SiO_2_: −43 mV; 15 nm SiO_2_: −48 mV; 30 nm SiO_2_: −55 mV; and 55 nm-SiO_2_: −50 mV. It was not possible to discern a clear trend that charge was affected by particle size.

For 9 nm, 15 nm, and 30 nm SiO_2_, very similar surface chemistry was recorded (determined by x-ray photoelectron spectroscopy [62]). Their specific surface reactivity (assessed by surface-induced biological oxidative damage and expressed as nM Trulox equivalent units per m^2^ nanomaterial [62]) was also identical within error. By contrast, the 55 nm SiO_2_ had a significantly different surface chemistry and, accordingly, also showed significantly different specific surface reactivity (half the reactivity of the other materials, but still significant above background) (Table 1).

### 2.2. Dose Setting and In Vitro Effective Concentration

The test material dose applied in the rat intratracheal instillation study (bolus dose of 360 µg/lung) was set to reflect the rat lung burden of 342 µg immediately after 6-day exposure to an aerosol concentration of 50 mg/m^3^ in the STIS [15]. (Of note, Maser et al. [59] presented ex vivo genotoxicity studies performed with lung and bone marrow cells from the rats submitted to the intratracheal instillation study described here and inadvertently reported that 5-day exposure to only 10 mg/m^3^ 15 nm SiO_2_ in the rat STIS yielded a lung burden of 342 µg.)

The test material concentration applied in the NR8383 AM assay ranged from 5.6 to 45 µg/mL, as pilot studies showed that this fully covered the whole range of effects (data not shown). Nominally, the highest concentration of 45 µg/mL could lead to a mean cell burden of 27 pg/cell (*cf.* Section 4.2 for calculation). For overall evaluation of the in vitro data, the particle mass–based concentrations (µg/mL) were multiplied with the respective test material’s surface area (m^2^/g, assessed by the Brunauer, Teller, and Emmett (BET) method), converting them to particle surface area–based concentrations (mm^2^/mL) (Table 2).

To estimate the effective concentration under cell culture conditions (affected by both particle gravitational settling and diffusion), the in vitro sedimentation, diffusion, and dosimetry (ISDD) model [66] was used. This was supplemented by data from analytical ultracentrifugation (AUC) dosimetry tests that allow measuring particle gravitational settling. The term *effective concentration* describes the particle mass per volume or particle surface area per volume–based dose (µg/mL and mm^2^/mL). Nevertheless, it is the proportion of the nominal *dose* (applied as homogeneous suspension) that reaches the bottom area of the wells and thus may have access to the cells that is relevant for the elicitation of cellular effects. The ISDD-based effective concentrations ranged from 15.8% for 55 nm SiO_2_ to 31.0% for 9 nm SiO_2_ (Table 2).

In the AUC dosimetry tests, the proportion of test materials (suspended in KRPG butter) that accumulated at the bottom of the vials within 24 h ranged from 4.3% for 55 nm SiO_2_ to 0.4% for 9 nm SiO_2_ (*cf.* Supplementary Information (SI), Table S1 and Figure S1). Values for colloidal SiO_2_ in other protein-free media are expected to lie in the same order of magnitude, since the particle sizes of the test materials were nearly identical in all protein-free media (Table 1). The AUC tests suggested that particle gravitational sedimentation to the bottom of the wells during the 16 h incubation period in the NR8383 AM assay was negligible. Hence, the effective concentrations of colloidal SiO_2_ appear to be dominated by diffusion. 

Importantly, the ISDD model [66], which addresses both particle sedimentation and diffusion, assumes a *“perfectly adsorptive (sticky) lower boundary condition”* [67], i.e., a probability of 1 that particles that come close to the cells adhere to them. Taking into account the pronounced negative charge of colloidal SiO_2_ (Table 1), particles might also diffuse to and from the bottom of the wells and the cells, so the probability of particle adherence might be much lower than 1. However, to the best of the authors’ knowledge, there is no test method that allows quantitative measurement of the fraction of colloidal SiO_2_ used in the present study that adhere to cultured cells. Therefore, the results presented below use ISDD-based effective concentrations even though the true availability of colloidal SiO_2_ is likely to be lower (*cf.* Section 3.1 for further discussion).

### 2.3. In Vitro NR8383 AM Assay

#### 2.3.1. H_2_O_2_ Synthesis

**Figure 1:**
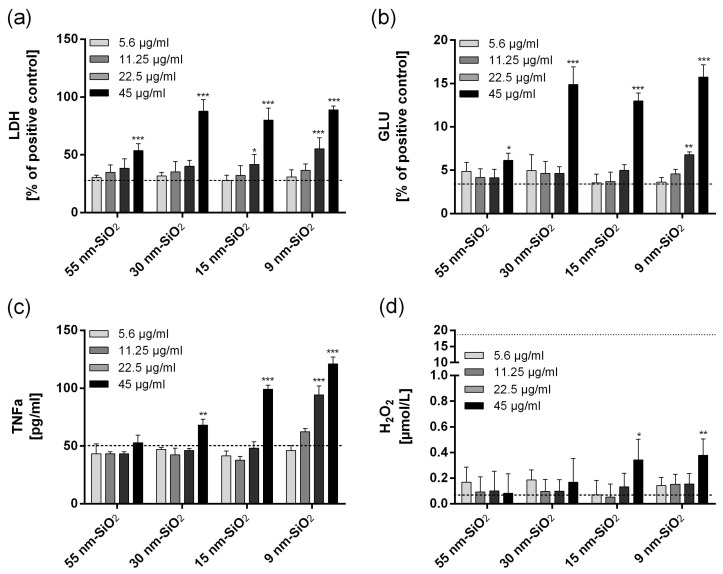
In vitro effects induced by 9 nm SiO_2_, 15 nm SiO_2_, 30 nm SiO_2_, and 55 nm SiO_2_ in the NR8383 alveolar macrophage (AM) assay. Cell culture supernatants were assessed for (**a**) lactate dehydrogenase (LDH, expressed relative to the positive control, 0.1% Triton X-100); (**b**) β-glucuronidase (GLU, expressed relative to the positive control); (**c**) tumor necrosis factor alpha (TNFα); and (**d**) H_2_O_2_. Values are expressed as means of three independent test runs ± standard deviation (* *p*-value ≤ 0.05; ** *p*-value ≤ 0.01; *** *p*-value ≤ 0.001). Levels of untreated vehicle control are indicated by dashed lines. For H_2_O_2_ measurement, the level reached upon application of 180 µg/mL zymosan (positive control) is indicated as a dotted line.

In the Amplex Red^®^ assay, 90 min incubation with 5.6–45 µg/mL 55 nm SiO_2_ or 30 nm SiO_2_ did not result in significant H_2_O_2_ synthesis. For the smaller 9 and 15 nm SiO_2_, moderately significant responses were observed at the highest concentration (45 µg/mL; *p*-values ≤ 0.01 and ≤0.05, respectively) (Figure 1 and SI; Table S2).

#### 2.3.2. LDH and GLU Release

For all four test materials, LDH release increased with increasing concentration (Figure 1 and SI; Table S2; *cf.* SI, Table S3 for test results expressed as x-fold change compared to corresponding vehicle controls). At the highest nominal concentration (45 µg/mL), all test materials elicited highly significant LDH release (*p*-value ≤ 0.001). Additionally, 9 nm and 15 nm SiO_2_ elicited moderately significant effects at 22.5 µg/mL (*p*-values ≤ 0.05 and ≤0.01, respectively). Hence, the two smaller test materials induced significant LDH release at lower concentrations than the two larger test materials. GLU release upon treatment with the four test materials largely reflected LDH release (Figure 1 and Table S2). However, it was less pronounced and the maximum values did not exceed 16% of GLU release elicited by the positive control 0.1% Triton X (Table S2).

As determined by TNFα-specific enzyme-linked immunosorbent assay (ELISA), TNFα content in the supernatant of the treated NR8383 AMs generally increased with increasing test material concentration. Further, at equal nominal concentrations of 22.5 and 45 µg/mL, TNFα release increased with decreasing particle size of the test materials. The 55 nm SiO_2_ did not elicit significant TNFα release at any concentration (5.6–45 µg/mL); 30 nm SiO_2_, 15 nm SiO_2_, and 9 nm SiO_2_ induced significant TNFα release at 45 µg/mL (*p*-values ≤ 0.01, ≤0.001, and ≤0.001, respectively). Furthermore, the 9 nm SiO_2_ induced significant TNFα release at 22.5 µg/mL (*p*-value ≤ 0.001) (Figure 1 and Table S2).

**Figure 2:**
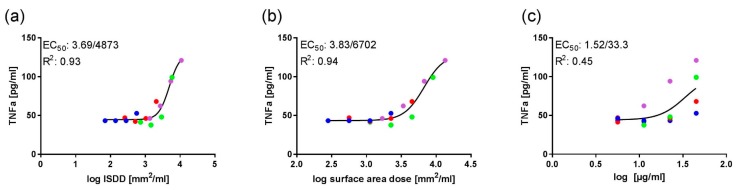
Combined evaluation of TNFα release induced by 9 nm SiO_2_, 15 nm SiO_2_, 30 nm SiO_2_, and 55 nm SiO_2_ in the NR8383 AM assay. Purple dots: 9 nm SiO_2_; green: 15 nm SiO_2_; red: 30 nm SiO_2_; blue: 55 nm SiO_2_. TNFα release was plotted by (**a**) particle surface area–based effective concentration (calculated by ISDD model), (**b**) particle surface area–based nominal concentration, and (**c**) nominal particle mass–based concentration (µg/mL). Plots were created with GraphPad Prism 7 applying the Levenberg-Marquardt method for nonlinear regression. EC_50_ values (test material concentrations inducing 50% increase in TNFα release as compared to vehicle control) are expressed as logarithmic/nonlogarithmic values (with the same units as the respective *x* axes).

Plotting the TNFα release data for all four test materials as surface area–based concentrations yielded sigmoidal curves (Figure 2a,b), with R^2^ values of 0.93 (ISDD-calculated effective concentrations) and 0.94 (nominal concentrations). EC_50_ values were 4873 mm^2^/mL and 6702 mm^2^/mL, respectively (Figure 2a,b). When the data were plotted as particle mass–based nominal concentrations, no such regularity was observed (Figure 2c). These plots indicate that effects were surface area–dependent (and hence also size-dependent).

#### 2.3.3. Overall Evaluation of In Vitro Test Results to Distinguish between Passive and Active Test Materials

Table 3 provides an overview of the in vitro lowest observed adverse effect concentrations (LOAECs, defined as the lowest test material concentration eliciting a significant cellular effect) recorded for 9 nm SiO_2_, 15 nm SiO_2_, 30 nm SiO_2_, and 55 nm SiO_2_ in the NR8383 AM assay. For each test material, the parameters (H_2_O_2_, LDH, GLU, or TNFα release) for which the in vitro LOAEC undercut the previously set threshold of 6000 mm^2^/mL [22] were recorded as positive. Test materials were assessed as active (MG4) if at least two parameters were positive, and passive (MG3) if no or only one parameter was positive (*cf.* Section 4.3.4 for further details on this two-out-of-four prediction model and on the setting of the 6000 mm^2^/mL threshold).

Applying the two-out-of-four prediction model to the *effective concentration*–based in vitro LOAECs, all four colloidal SiO_2_ were assessed as active (MG4): for 55 nm SiO_2_, the in vitro LOAECs recorded for LDH and GLU release undercut the 6000 mm^2^/mL threshold (two parameters positive); for 30 nm SiO_2_, the in vitro LOAEC recorded for TNFα release (three parameters positive); and for 9 nm SiO_2_ and 15 nm SiO_2_, all four in vitro LOAECs undercut the 6000 mm^2^/mL threshold (four parameters positive) (Table 3).

In contrast, applying the two-out-of-four prediction model to the *nominal concentration*–based in vitro LOAECs, the same overall outcome was obtained for only 30 nm SiO_2_ and 55 nm SiO_2_ (indicating activity (MG4)), whereas 9 nm SiO_2_ and 15 nm SiO_2_ were assessed as passive (MG3) (15 nm SiO_2_: only LDH positive; 9 nm SiO_2_: all four parameters negative) (Table 3).

#### 2.3.4. In Vivo Rat Intratracheal Instillation Study

**Figure 3:**
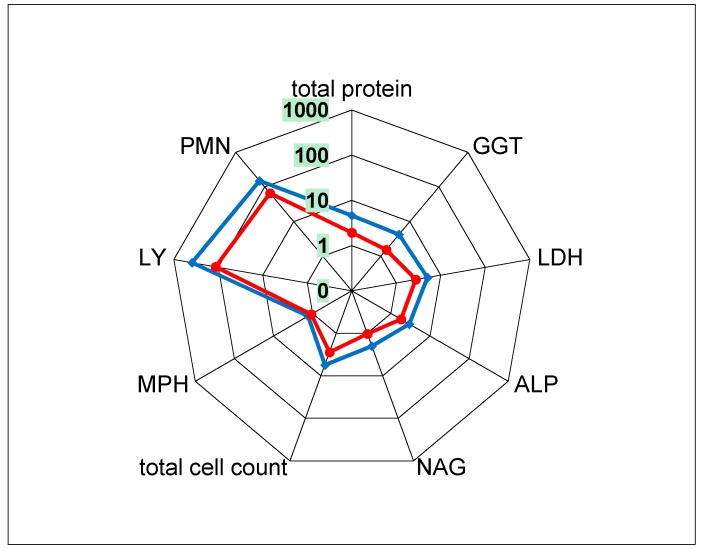
Cell counts, total protein, and enzymes in bronchoalveolar lavage fluid of rats 72 h after single intratracheal instillation of 360 µg 55 nm or 15 nm SiO_2_ (from Maser et al. [59]). Blue line: 15 nm SiO_2_; red: 55 nm SiO_2_. Abbreviations: ALP: alkaline phosphatase; GGT: γ-glutamyltransferase; LDH: lactate dehydrogenase; LY: absolute cell count for lymphocytes; MPH: absolute cell count for macrophages; PMN: absolute cell count for polymorphonuclear neutrophilic granulocytes; NAG: *N*-acetyl-β-glucosaminidase.

Before and after instillation, rats treated with single bolus doses of 360 µg 15 nm or 55 nm SiO_2_ per lung showed no clinical signs that differed from the control group. Mean body weights measured before instillation, on the first day after instillation, and on day three just before necropsy were also comparable to the control animals (Table 4). The hematological examination revealed a small but significant decrease in platelet counts for 55 nm SiO_2_ and a significant increase in absolute neutrophil counts for 15 nm SiO_2_ (Table 5 and Figure 3). In the bronchoalveolar lavage fluid (BALF) of rats treated with 55 nm SiO_2_, significant increases in absolute lymphocytes, polymorphonuclear neutrophils (PMNs), atypical cells, and eosinophils were recorded. In the BALF of rats treated with 15 nm SiO_2_, the absolute counts of all examined cell types (except for macrophages) were significantly increased. The increased total cell count was mainly attributed to the influx of PMNs and lymphocytes. Consistently, the cytological alterations recorded in the BALF of rats treated with 15 nm SiO_2_ were qualitatively similar but more pronounced than those induced by 55 nm SiO_2_. Further, the total protein level in the BALF of rats treated with 15 nm SiO_2_ was elevated 4.7-fold compared to the control animals, and all measured enzyme activity (γ-glutamyltransferase (GGT), LDH, alkaline phosphatase (ALP), and *N*-acetyl-β-glucosaminidase (NAG)) was increased two- to fivefold. In the BALF of rats treated with 55 nm SiO_2_, the total protein level increased 1.9-fold, and LDH and ALP were the only enzymes with significantly increased activity (2.7 and 1.8-fold, respectively) (Table 5 and Figure 3).

The observation that 15 nm SiO_2_ elicited more pronounced effects than 55 nm SiO_2_ was further underlined by the pathological findings (Table 4). Rats treated with 15 nm SiO_2_ showed noticeable increases in lung weight (+38% compared to the control group) and spleen weight (+19%). Furthermore, they exhibited enlarged mediastinal lymph nodes, most likely due to inflammatory activation. By comparison, treatment with 55 nm SiO_2_ led to no such abnormalities. For both treatment groups, the increased lung weight was assessed as being caused by an influx of inflammatory cells and the resulting swelling of the tissue due to overall inflammation, and they were consistent with the histopathological examination: in all lung lobes of two of the three animals treated with 55 nm SiO_2_, mild multifocal granulomatous inflammation with thickening of the alveolar walls was observed, caused by infiltrated macrophages and granulocytes. Again, the lungs of all three rats from the 15 nm SiO_2_ treatment group were more severely affected, and mild to severe lympho-reticular hyperplasia of the mediastinal lymph nodes was recorded (data not shown). This microscopic finding is common when lymph nodes are activated by an inflammatory process in the area of drainage, and it was consistent with the macroscopically diagnosed lymph node enlargement (Maser et al. [59]).

The pulmonary effects elicited by 15 nm SiO_2_ 3 days after intratracheal administration of a single bolus dose of 360 µg/lung were more pronounced than the respective findings in a rat STIS at comparable lung burden (i.e., 342 µg shortly after 5-day inhalation exposure to 50 mg/m^3^ 15 nm SiO_2_ [15]; Box 2). Possibly the high dose rate induced by intratracheal instillation compared to the continuous lower dose rate in the STIS during aerosol administration accounts for the observed difference in pulmonary findings.

Box 2 **Rat short-term inhalation toxicity of 15 nm-SiO_2_ [15].**

In a rat STIS (aerosol administration for 5 consecutive days; 6 h/day) [15], inhalation exposure to 50 mg/m^3^ 15 nm-SiO_2_ caused a lung burden of 342 µg immediately after the 5-day inhalation exposure period and marginal systemic inflammation, evidenced by slight and transient increases in granulocyte counts in the blood. Increased PMN and lymphocyte counts were present in the BALF of this high concentration test group shortly after exposure and (in the 10 and 50 mg/m^3^ test groups) 3-weeks post-exposure. Histologically, multifocal macrophage aggregates were observed in the lung shortly after exposure. This finding exacerbated towards a slight multifocal pulmonary inflammation by the end of the 3-week post-exposure period. The NOAEC of 15 nm-SiO_2_ was assessed as 2.5 mg/m^3^ [15]. Applying the DF4nanoGrouping threshold (STIS NOAEC < 10 mg/m^3^ = (MG4) active nanomaterial), 15 nm-SiO_2_ was assigned as (MG4) active [33].

### 2.4. Summary and In Vitro–In Vivo Correlation of the Test Results

In the in vitro NR8383 AM assay, all four test materials elicited a concentration-dependent release of LDH, GLU, and TNFα (between 5.6 and 45 µg/mL). Further, the two smaller test materials (9 and 15 nm SiO_2_) induced moderate but significant H_2_O_2_ release at the highest nominal concentration (45 µg/mL). As can be seen in Figure 1 and SI, Table S2, the severity of in vitro findings increased with increasing specific surface area of the colloidal SiO_2_ (and hence decreasing particle size).

**Figure 4:**
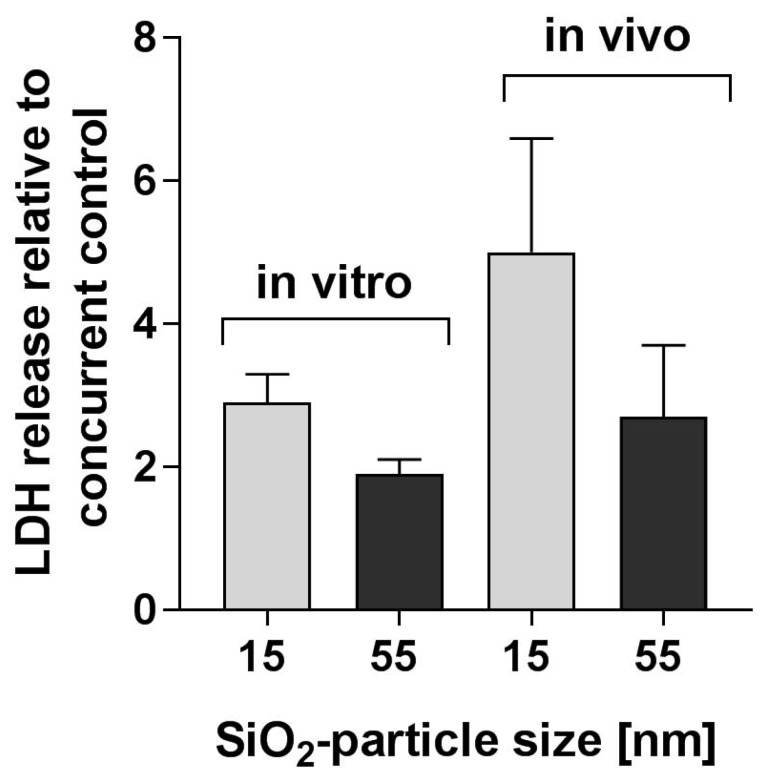
Comparison of in vitro LDH release (NR8383 assay) and in vivo LDH release (rat intratracheal instillation study) elicited by 15 nm and 55 nm SiO_2_. In vitro LDH release was elicited by 45 µg/mL of either 15 nm or 55 nm SiO_2_ in the NR8383 assay; in vivo LDH release was elicited by a bolus dose of 360 µg/lung (intratracheal instillation). The in vitro test material dose (nominally 30 pg/NR8383 AM or 45 µg/mL) was selected to reflect the in vivo dose (nominally 27–36 pg/AM or 360 µg/lung).

Since LDH release, which mirrors cell membrane damage, was determined in the NR8383 AM assay and in the BALF of rats submitted to the intratracheal instillation study, the in vitro and in vivo data obtained for this endpoint were compared (Figure 4). To facilitate in vitro–in vivo comparison, the highest nominal in vitro concentration (45 µg/mL) and the in vivo dose (360 µg/lung) were selected to reflect similar material mass per AM (in vitro: 30 pg/NR8383 AM; in vivo: 36–27 pg/AM in rat lungs; *cf.* Section 4.2). Both in vitro and in vivo, LDH release was more pronounced for the 15 nm SiO_2_ than for the 55 nm SiO_2_. Further, for both test materials, the in vitro effects were less pronounced than the in vivo effects, albeit with overlapping standard deviations (Figure 4). Due to the limited database (two test materials), this estimation was not verified by statistical analysis.

When the two-out-of-four prediction model was applied, expressing test results as surface area–based effective concentrations (estimated by ISDD modelling [66]), all four test materials were assigned as active (MG4) nanomaterials (Table 3). (When expressing test results as nominal concentrations, only 30 nm and 55 nm SiO_2_ were assessed as active (MG4).) The available in vivo data support the overall appraisal of colloidal SiO_2_ as active, and hence the use of effective concentrations for the overall evaluation: Applying the DF4nanoGrouping STIS-related threshold (MG4 indicated by a STIS NOAEC below 10 mg/m^3^ [32,33]), 15 nm SiO_2_ was assigned as active (MG4) (STIS NOAEC: 2.5 mg/m^3^ [15]; Box 2). STIS data are unavailable for the other three test materials. While the DF4nanoGrouping does not include an evaluation scheme for intratracheal instillation data, the pulmonary effects elicited by 55 nm and 15 nm SiO_2_ in the intratracheal instillation study strongly support the in vitro assessment that these test materials are active (MG4).

## 3. Discussion

The present study aimed at assessing four extensively characterized colloidal SiO_2_ (PPS: 9 nm, 15 nm, 30 nm, and 55 nm) by the NR8383 AM assay and comparing the in vitro findings to in vivo pulmonary effects elicited by 15 nm and 55 nm SiO_2_ in a rat intratracheal instillation study (3 days after administration of a single bolus dose) and available rat STIS data for 15 nm SiO_2_. The study served the overarching goal of identifying intrinsic material properties and system-dependent properties of colloidal SiO_2_ that may affect its in vitro and in vivo pulmonary toxicity (further discussed in Section 3.1) and further elucidating the applicability of the NR8383 AM assay published by Wiemann et al. [22] in predicting the pulmonary toxicity of well-dispersed colloidal amorphous SiO_2_. Section 3.2 discusses the test materials’ in vitro effective concentrations and Section 3.3 their in vitro effects on NR8383 AMs; Section 3.4 further assesses the in vitro effects of different amorphous SiO_2_, and Section 3.5 provides an in vitro–in vivo comparison of the findings.

### 3.1. Relevant Intrinsic Material and System-Dependent Properties of Colloidal Amorphous SiO_2_

All four test materials were extensively characterized to yield data for all grouping criteria of both tiers of the DF4nanoGrouping (Box 1): the intrinsic material properties water solubility, particle morphology, and chemical composition (Tier 1), and the system-dependent properties dissolution rate in biological media, surface reactivity, and particle dispersibility (Tier 2) [32,33].

Water solubility of the test materials decreased with increasing PPS (from 48 mg/L for 9 nm SiO_2_ to 7 mg/L for 55 nm SiO_2_; Table 1) and remained well below the DF4nanoGrouping threshold of 100 mg/L [32,33]. Consistent with these findings, a low dissolution rate in phagolysosomal simulant fluid was recorded that was again inversely proportional to PPS (Table 1). In consequence, none of the test materials were assigned as soluble (MG1) nanomaterials.

All four colloidal SiO_2_ remained well dispersed in F-12K medium, KRPG buffer, and 0.9% NaCl solution (Table 1). The pronounced dispersibility in protein-free media is highly relevant for in vitro dosimetry. Further, within the DF4nanoGrouping, high dispersibility indicates potential in vivo mobility, resulting in a precautionary assignment of such nanomaterials as active (MG4) [32,33]. However, high dispersibility in protein-free media may overestimate intrapulmonary mobility. Notably, 15 nm and 55 nm SiO_2_ strongly agglomerated in DMEM + FCS (Table 2), indicating nonmobility, whereas dispersibility was not assessed in other protein-containing fluids that more closely resembled the in vivo lung lining fluid.

The specific surface reactivity of colloidal amorphous SiO_2_ (assessed by surface-induced biological oxidative damage and expressed as nM Trulox equivalent units per m^2^ nanomaterial) was significantly above zero. Nevertheless, for all four colloidal SiO_2_ it was consistently below 10% of the reactivity of strongly oxidative Mn_2_O_3_ nanoparticles. Hence, the specific surface reactivity of colloidal SiO_2_ did not indicate activity (MG4) according to this DF4nanoGrouping threshold [32,33].

In contrast to the NR8383 AM assay (and other in vitro test methods), abiotic test methods to determine surface reactivity (e.g., surface-induced biological oxidative damage, electron spin resonance, or ferric-reducing ability of serum) do not require gravitational sedimentation of the test materials but are performed under constant stirring. Therefore, abiotic test methods might provide relevant data to prevent false negative in vitro test results for materials that are actually active (MG4) but too well dispersed to reach the bottom of the wells or the cultured cells. Despite this, the results from the present study show that the cellular effects elicited by colloidal SiO_2_ in the NR8383 AM assay were not predominantly elicited by its oxidative surface reactivity.

Based on evaluation of the DF4nanoGrouping Tier 1 and Tier 2 grouping criteria [32,33] the four colloidal SiO_2_ are assigned as active (MG4) on account of their dispersibility (and the cellular effects observed in the NR8383 AM assay, as further discussed in Section 3.3).

### 3.2. In Vitro Effective Concentration

In Wiemann et al. [22], which first described the NR8383 AM assay, the vast majority of test materials underwent rapid gravitational settling and could therefore be engulfed by the NR8383 AMs. Accordingly, the effective concentration of these materials was considered to be consistent with their nominal (total applied) concentration. This provided a good starting point for the overall successful in vitro–in vivo comparison, and for the well-dispersed nonsettling 15 nm SiO_2_, even though those authors did not correct the corresponding in vitro results for the effective concentration (*cf.* Table 3 of the present study, bottom row).

In the present study, the four colloidal SiO_2_, which differed only by PPS, elicited concentration- and inversely size-dependent effects in the NR8383 AM assay. To account for the high dispersibility of the test materials in interpreting the in vitro findings, the in vitro effective concentrations were estimated. Based on ISDD modelling [66], the effective concentrations ranged from 15.8% for the 55 nm SiO_2_ to 31.0% for the 9 nm SiO_2_. Hence, the transport prediction by ISDD is dominated by diffusion. By comparison, the AUC measurements, which suppress the contribution of diffusion transport and determine only sedimentation transport, overall indicated only minimal gravitational settling of all tested colloidal amorphous SiO_2_ in protein-free media. However, an approximately 10-fold more pronounced sedimentation was recorded for the 55 nm SiO_2_ (4.3% after 24 h) than for the 9 nm SiO_2_ (0.4%) (SI, Table S1 and Figure S1). In contrast to particle sedimentation (larger particles sediment more quickly than smaller ones), particle diffusion is inversely proportional to particle size. The observation that the smaller colloidal SiO_2_ elicited more pronounced in vitro effects than the larger might be interpreted as higher effective dose of the smaller sized particles due to their transport by diffusion.

However, the in vitro effective concentration of nanomaterials is also influenced by the proportion of nanoparticles that adhere to the bottom of the wells, i.e., gathered within the reach of the cultured cells [56,57,58,66,67]. Since the ISSD model assumes a “sticky” lower boundary condition, i.e., a probability of 1 that nanoparticles that come close to the cultured cells adhere to them, and since both colloidal SiO_2_ and cell surfaces are negatively charged, it might overestimate the effective concentration, possibly to different extents for different particles [67]. The one-dimensional distorted grid (DG) model allows adjusting the probability for nanoparticle adherence by including either a “sticky” or “reflective” bottom assumption [67,68]. When the sticky bottom assumption is selected, every particle that touches the bottom of the well due to diffusion is counted as sedimented. This results in a greater weighting of movement by diffusion during the modelling. By contrast, when the reflective bottom assumption is selected, particles that touch the bottom of the well are not counted as sedimented [68]. DG modelling was performed to further investigate how the probability of particle adherence to the bottom of the wells affected the in vitro effective concentrations of the four colloidal SiO_2_. The DG model–based effective concentrations matched the ISDD model–based predictions in terms of both absolute levels and size-dependent ranking of the colloidal SiO_2_ (Table 2) if the sticky bottom assumption was selected (SI, Figure S2A). If the DG model was applied using the reflective bottom assumption [68], the AUC measurements of the sedimented proportion of test materials (SI, Figure S1) matched the DG model–based effective concentrations in terms of both absolute levels and size-dependent ranking of the colloidal SiO_2_ (SI, Figure S2B).

Based on these calculations, and further considering that a concrete value for the stickiness of an NR8383 AM culture is not yet available, it was decided to use the ISDD-based estimations (and hence the sticky bottom assumption) to evaluate the in vitro results in spite of limitations of the ISDD model in calculating effective concentrations of colloidal SiO_2_ highlighted by the developers of the DG model (Phil Demokritou, Harvard University, Cambridge, MA, USA; personal communication, 2017).

By contrast, the DG model–based estimations applying the reflective bottom assumption (and, concordantly, the AUC measurements) were assessed as underrating the “true” effective concentrations. Considering that all four colloidal SiO_2_ elicited concentration-dependent cellular effects, effective concentrations ranging below 1% (at 16 h) would point to very toxic test materials. However, the available in vivo studies provide no indication for such high toxicity. Taken together, these observations can be interpreted as indicating that the probability of particles reflecting from the direct vicinity of cells is neither 0 nor 1, but somewhere in between. Presumably, the “true” effective concentration of the test materials in the NR8383 AM assay ranged between the (DG or ISDD model–based) sticky bottom and reflective bottom assumption estimations (and hence the AUC data). In this regard, it would be important to consider and include the highly evolved particle uptake mechanisms of macrophages into realistic models predicting the in vitro effective dose.

In summary, these observations highlight the need to consider in vitro dosimetry when in vitro test systems are used to assess nanomaterials of high dispersion stability, but also the challenges that such considerations entail. This caveat applies especially to colloidal SiO_2_ when dispersed in protein-free media, but does not, in general, extend to pyrogenic or precipitated SiO_2_, which has much lower dispersion stability. To the best of the authors’ knowledge, a test method that allows *quantitatively* determining the proportion of nanoparticles making up the effective dose, i.e., within and/or upon the cells, is not yet available. For specifically designed nanomaterials, stimulated emission depletion microscopy might be a suitable method to enable such measurements [69]. Further, fluorescence labelling of the test materials enables *qualitatively* investigating in vitro cellular uptake. Previous studies with fluorescent colloidal SiO_2_ showed a fluorescent halo at the outer membrane of NR8383 AMs (data not shown), suggesting that NR8383 AM cells may indeed provide a sticky surface structure for colloidal SiO_2_. Such a structure might enhance cellular uptake of the test materials, and hence elicitation of cellular effects (*cf.* also Section 3.4).

When estimating in vitro effective concentrations, the assumptions underlying the calculations (in terms of particle sedimentation, diffusion, and adherence to the cells) should be specified and the strengths and limitations of the applied model identified. The ensuing uncertainties related to the calculated effective concentrations should be addressed in the evaluation of in vitro test results [70]. For the NR8383 AM assay specifically, whenever the two-out-of-four prediction model yields different results using nominal concentration–based or effective concentration–based results, the calculated effective concentration–based in vitro LOAECs should be used for the overall evaluation. At present, the ISDD model [66] appears to provide a reasonable upper estimate of the effective concentration, yielding a conservative approach for hazard and risk assessment.

### 3.3. In Vitro Effects of Colloidal Amorphous SiO_2_

All four test materials clearly elicited concentration-dependent release of LDH, GLU, and TNFα in the NR8383 AM assay, and the two smaller test materials further elicited significant H_2_O_2_ release at the highest nominal concentration (45 µg/mL; SI, Table S2). This observation that the smaller 9 nm and 15 nm SiO_2_ elicited more pronounced in vitro effects than the larger 55 nm and 30 nm SiO_2_ is consistent with the outcome of the in vivo intratracheal instillation study, where 15 nm SiO_2_ elicited more severe pulmonary effects than 55 nm SiO_2_ (Table 4 and Table 5).

When TNFα release was plotted jointly for all four colloidal SiO_2_, more stringent dose-response curves were obtained when the effective (ISDD-modelled) and nominal test material concentrations were expressed in surface area–based metrics (mm^2^/L; Figure 2). Interestingly, this was not obtained for LDH or GLU release (data not shown). This suggests that TNFα release is attributable to the test material’s surface properties, rather than mass, whereas LDH or GLU release partly or entirely depends on other intrinsic or system-dependent properties. TNFα induction upon in vitro administration of amorphous SiO_2_ is known to involve particle uptake as well as secondary adenosine signaling and NLRP3 proteasome activation [71].

In the present study, the amount of released TNFα was measured by ELISA. By comparison, Wiemann et al. [22] used the L929 cytolysis test [72] to measure the bioactive TNFα induced in the supernatant of the treated NR8383 AMs. ELISA was considered preferable, as it rules out direct cellular effects of nonsedimented colloidal SiO_2_ nanoparticles on L929 cells.

For overall evaluation of the test results from the NR8383 AM assay, Wiemann et al. [22] set a threshold of 6000 mm^2^/mL to distinguish between test material–specific effects and effects caused by unspecific cellular overload. This threshold was calculated as being the highest particle surface area–based concentration that does not yet result in particle overload of the NR8383 AMs. The threshold was expressed in surface area–based metrics, since the effects of nanomaterials, when applied under non–cellular-overload conditions, appear to be mainly conveyed by particle surface, not particle mass [45,73,74,75]. Rushton et al. [19] showed that in vitro studies with AMs (assessing ROS generation) correlated significantly with in vivo rat intratracheal instillation studies (measuring PMN counts 24 h after instillation) when biological activity was expressed by particle surface area metrics.

Using *effective concentration*–based in vitro LOAECs in the two-out-of-four prediction model (described by Wiemann et al. [22] for overall evaluation of the outcome of the NR8383 AM assay), all four test materials were assessed as active (MG4) (Table 3). In contrast, when *nominal concentration*–based in vitro LOAECs were used, only the two larger test materials (30 nm and 55 nm SiO_2_) were assessed as active (MG4), whereas 15 nm and 9 nm SiO_2_ exhibited effects only above the threshold of 6000 mm^2^/mL, indicating cellular overload (i.e., in vitro LOAECs of 6750 mm^2^/mL for both LDH and GLU release; Table 3). These nominal concentration–based results might also be interpreted as borderline non–cellular overload, and therefore inconclusive, effects. As highlighted by Leontaridou et al. [70], in vitro test methods (just as any other test method) inherently have a borderline range around their classification threshold within which test results are inconclusive due to the test method’s biological and technical variability.

In summary, the particle surface area–based threshold of 6000 mm^2^/mL [22] proved useful to assess the activity of colloidal SiO_2_ using ISDD-modelled effective concentration–based test results. Nevertheless, as further knowledge becomes available on how the intrinsic material properties of nanomaterials affect their in vitro and in vivo toxicity, this surface area–based threshold and the two-out-of-four prediction model may need to be refined and adapted to take into account further material properties that may affect the in vitro or in vivo effects of specific types of nanomaterials.

### 3.4. Further In Vitro Studies Investigating Colloidal SiO_2_ and Other Amorphous SiO_2_

In the NR8383 AM assay, protein-free media (i.e., F-12K medium and KRPG buffer) are used to prevent particle agglomeration. By contrast, media containing, e.g., lipids, surfactant proteins, or serum proteins destabilize colloidal test materials, resulting in particle agglomeration [55,76,77,78,79]. The degree of agglomeration affects the nanoparticles’ sedimentation in vitro (*cf.* Section 3.2), and hence the manner in which they come into contact with the cultured cells [80]. However, the cellular uptake of colloidal SiO_2_ nanoparticles also appears to depend on the type of cultured cells [81], and noninternalized particles can also damage the cell membrane, and hence induce cellular effects, by diffusion contact [74,82].

Further, numerous studies have concluded that the in vitro toxicity of amorphous SiO_2_ nanomaterials is mitigated in the presence of serum supplements [6,83,84,85,86]. Despite this, when prepared in cell culture media supplemented with 10% FCS, the four colloidal amorphous SiO_2_ tested in the present study induced different forms of genotoxicity in the alkaline Comet assay using V79 cells or precision-cut rat lung slices and in the alkaline unwinding assay using A549 cells [59]. Using EpiAirway^TM^ 3D human bronchial models maintained in EpiAirway^TM^ culture medium not supplemented with serum (*cf.* https://www.mattek.com/products/epiairway/), 15 nm SiO_2_ (as used in the present study) and 15 nm SiO_2_ with phosphonate surface functionalization induced marginal but still significant genotoxicity in the alkaline Comet assay at 50 µg/cm^2^ tissue, but no cytotoxicity in either the LDH or adenosine triphosphate assay [87]. However, ex vivo, neither 15 nm nor 55 nm SiO_2_ caused genotoxic effects in lung or bone marrow cells [59] from rats submitted to the intratracheal instillation study described here.

In addition, 15 nm and 55 nm SiO_2_ were assessed in a 3D reconstructed skin micronucleus assay using EpiDerm^TM^ tissue maintained in the supplier’s growth medium [88], also not supplemented with serum (*cf.* https://www.mattek.com/products/epiderm/). Test material cellular uptake analysis revealed no exposure of 15 nm or 55 nm SiO_2_ to the EpiDerm^TM^ cells upon topical application (and hence no cytotoxicity or genotoxicity) and confounding barrier effects of the collagen cell attachment layer during in-medium exposure [88]. By contrast, 15 nm and 55 nm SiO_2_ were extensively taken up into two-dimensional TK6 cell cultures (cultured in RPMI 1640 medium + 10% horse serum), where they caused genotoxicity and cytotoxicity [88].

While the NR8383 AM assay does not use serum supplements, nanoparticles in the lung may become coated with proteins/and or lipids from the lung lining fluid over time, and this can facilitate nanoparticle agglomeration inside the alveoli. The degree of agglomeration in lung surfactants likely influences nanoparticle uptake into AMs, and thereby also nanoparticle clearance and toxicity [55]. In spite of the substantial differences between the in vivo lung and the NR8383 AM test system, the serum-free approach of the NR8383 AM assay has proven useful in distinguishing passive (MG3) from active (MG4) nanomaterials [22]. The present study adds further evidence confirming the usefulness of the NR8383 AM assay for assessing colloidal SiO_2_, while highlighting the need to consider the effective concentration of such test materials with limited gravitational settling.

### 3.5. In Vitro–In Vivo Correlations of the Test Results

For four differently sized but otherwise identical colloidal SiO_2_, the present study yields quantitative in vitro data for four parameters (LDH, GLU, H_2_O_2_, and TNFα release) and, for two of the test materials, quantitative in vivo data for a spectrum of BALF parameters (differential cell counts, total protein, and LDH, GGT, ALP, and NAG activity). Although the highest in vitro and in vivo nominal doses per AM were similarly in the range of 27–36 pg/AM (*cf.* Section 4.2), the differences in the in vitro and in vivo test designs (e.g., with respect to dose rate, time point of recording post-application, fluid volume) must be considered when comparing the results. Regardless of such differences, both the in vitro and in vivo effects elicited by 15 nm SiO_2_ were generally more pronounced than those induced by 55 nm SiO_2_ (Figure 1 and Figure 3; Table 4 and Table 5). Therefore, the relative differences between the in vivo and in vitro effects of these two test materials (at nominal concentrations of 45 µg/mL) were further evaluated, revealing that the relative differences (expressed as fold increase induced by 15 nm SiO_2_ compared to 55 nm SiO_2_) were very similar for a broad spectrum of parameters:

- In vitro: TNFα: 1.9-fold; GLU: 2.1-fold; H_2_O_2_: 3-fold.
- In vivo (BALF): ALP: 1.6-fold; NAG: 2-fold; total protein: 2.4-fold; GGT: 2.8-fold; inflammatory cells: 1.2-fold to 2.5-fold (Table S2 and Table 5). Only the increase of eosinophils in BALF was exceptionally high (10-fold). This alteration could not be explained.

A direct in vitro and in vivo comparison is shown for LDH relative to the respective controls (Figure 4). Taking into account the standard deviation of these values, the relative differences of the LDH release in vitro and in vivo induced by 15 nm and 55 nm SiO_2_ were very similar, although apparently more pronounced in BALF from the lungs of test material–treated rats (Figure 4). A reason for this difference might be that, as outlined above, the in vitro effective concentration may differ from the nominal concentration, whereas inside the lung all particles will contact lung parenchymal structures, i.e., the alveolar inner surface, once the instillation fluid is resorbed. Therefore, the effective dose upon intratracheal instillation was most likely identical with the nominal dose, and differences in the in vivo short-term pulmonary effects induced by 15 nm and 55 nm SiO_2_ can be attributed to the fourfold difference in the BET-specific surface area between the two (200 m^2^/g and 50 m^2^/g, respectively; Table 1).

The differences between in vitro and in vivo LDH release might also be explained by differences in the dose rates of the different test methods. In vivo, differences in dose rates have been shown to affect the severity of pulmonary findings [89]. Generally, at the same lung burden, the dose rate is higher in intratracheal instillation studies (bolus dose application) than in inhalation studies (longer-term inhalation exposure). Similarly, at the same burden per AM, the dose rate in the intratracheal instillation study, where a small volume of test material preparations reached the much larger surface of the lung almost immediately, was much higher than in the NR8383 AM assay, where the particles predominantly reached the cells by diffusion over a longer period of time (i.e., the 16 h incubation period). Further, in vivo, LDH can be released not only by AMs, but also by other pulmonary cells (e.g., epithelial cells), and the microenvironment of the airway lumen has a considerable influence on many aspects of AM function [90]. By contrast, the in vitro test system used in the NR8383 AM assay only includes AMs. Finally, LDH has been observed to bind onto the surface of nanomaterials in protein-free media, adulterating the detection of test material–induced cell membrane damage [91].

When the two-out-of-four prediction model was applied for overall evaluation of the outcome of the NR8383 AM assay expressing test results as surface area–based effective concentrations (estimated by ISDD modelling), all four test materials were assigned as active (MG4) nanomaterials (Table 3). This is consistent with the available rat STIS data yielding a NOAEL of 2.5 mg/m^3^ for 15 nm SiO_2_ [15], on account of which this material was assigned as active (MG4), applying the DF4nanoGrouping Tier 3 threshold of STIS NOAEC <10 mg/m^3^ [32,33]. While that grouping does not include an evaluation scheme for intratracheal instillation data, the pulmonary effects elicited by 55 nm and 15 nm SiO_2_ in the intratracheal instillation study strongly support the in vitro assessment that these test materials are active (MG4).

Notably, the assignment of colloidal amorphous SiO_2_ as active (MG4) is relevant for the inhalation route of exposure, but this assessment cannot be extrapolated to other routes of exposure, or to other types of amorphous SiO_2_. Buesen et al. [92] did not record any treatment-related adverse effects of 15 nm SiO_2_ (without or with different surface functionalizations) in rats upon 28-day oral administration (OECD TG 407). Hofmann et al. [93] and Wolterbeek et al. [94] observed no reproductive or developmental toxicity of precipitated SiO_2_ in rats (OECD TG 414 and 416, respectively); and Kolle et al. [95] reported no in vitro eye-irritating potential of either precipitated or pyrogenic SiO_2_ in the bovine corneal opacity and permeability assay (OECD TG 437) or the reconstructed human cornea–like epithelium test method (OECD TG 492).

In DF4nanoGrouping case studies [33], a precipitated amorphous SiO_2_ and a pyrogenic amorphous SiO_2_ were assigned as borderline (MG1) soluble nanomaterials, since they were partially soluble in water and DMEM supplemented with 10% FCS, but highly soluble in Gamble’s solution. By contrast, 15 nm SiO_2_ was not water soluble and did not dissolve in biological media, and it was assigned as active (MG4) on account of its in vitro cellular effects, confirmed by the Tier 3 rat STIS data. Further, four surface-functionalized variants of 15 nm SiO_2_ were assessed; two were assigned as passive (MG3) and two as active (MG4) nanomaterials

In summary, the high degree of coincidence between the in vitro and in vivo results obtained for 15 nm and 55 nm SiO_2_ underlines the predictive capacity of the NR8383 AM assay. Nevertheless, the pathophysiology of the lung is clearly far more complex, since many more cell types are compromised, as is reflected by the release of cell type–specific enzymes and the presence of invading cells, such as PMNs and eosinophils, in BALF.

## 4. Materials and Methods

### 4.1. Test Materials and Test Material Characterization

The test materials 9 nm SiO_2_, 15 nm SiO_2_, 30 nm SiO_2_, and 55 nm SiO_2_ were produced as 30%, 40%, 45%, and 50% suspensions, respectively, by AkzoNobel AB (Bohus, Sweden; formerly H.C. Starck GmbH, Leverkusen, Germany). The particle size distribution was determined by AUC or dynamic light scattering, as described by Wohlleben [41] and Wohlleben et al. [96]. To determine water solubility, forced filtration through 5 kDa filters at pH 7 was applied [60,97]. For this purpose, the original test material suspensions were diluted to 10 mg/mL to prevent filter clogging. Dissolution rate was determined as described by Wohlleben et al. [98]. Surface chemistry and specific surface reactivity were determined by X-ray photoelectron spectroscopy and surface-induced biological oxidative damage, respectively, as described by Gandon et al. [62]. Table 1 provides further details on the test material characterization.

### 4.2. Dose Setting and In Vitro Effective Concentration

The highest test material concentration applied in the NR8383 AM assay and dose applied in the rat intratracheal instillation study were calculated to reflect an aerosol concentration of 50 mg/m^3^ in the rat STIS. In the rat STIS, inhalation exposure to 50 mg/m^3^ 15 nm SiO_2_ for 6 h a day on 5 consecutive days resulted in a lung burden of 342 µg [15]. Accordingly, for the intratracheal instillation study, a bolus dose of 360 µg/lung was selected.

In order to transfer the in vivo dose to the in vitro dose, the test material content per AM was calculated. The macrophage population per rat lung ranges between 10 × 10^6^ and 13 × 10^6^ cells for rats weighing 200–300 g [25,99,100]. Considering a consistent, complete deposition of instilled material and complete uptake by macrophages, 360 µg 15 nm SiO_2_ per lung corresponds to 36–27 pg test material per AM. To meet this concentration range, 30 pg/AM was selected as the highest nominal dose in the in vitro NR8383 AM assay. Calculated with a volume of 200 μL/well and a defined population of 3 × 10^5^ cells/well, this corresponds to a concentration of 45 μg/mL. Accordingly, 45 µg/mL was selected as the highest test material concentration in the NR8383 AM assay.

For data evaluation, particle mass–based test material concentrations were converted to particle surface area–based concentrations identified as most suitable for the NR8383 AM assay [22]. For this purpose, the applied particle mass–based concentrations (µg/mL) were multiplied with the respective test material’s surface area (m^2^/g) as assessed by the BET method. This conversion resulted in the dose metric of particle surface area per volume (mm^2^/mL) (Table 2).

The in vitro effective concentration was calculated using the ISDD model [66] with the following parameters: dish depth: 0.006 m; volume: 0.2 mL; viscosity (H_2_O, 37 °C): 0.00074 mPa s; temperature: 310 K; SiO_2_ density: 2.3 g/mL; particle size in F-12K medium (*cf.* Table 1) equals agglomerate size; incubation time: 16 h; packing factor: 0.64; fractal dimension: 42,796.00. Test material sedimentation was assessed by AUC as described by Sauer et al. [78].

### 4.3. In Vitro NR8383 AM Assay

NR8383 AMs (ATCC, USA; ATCC^®^ No.: CRL-2192TM) were maintained in F-12K cell culture medium (Sigma-Aldrich, Taufkirchen, Germany) supplemented with 15% fetal calf serum (FCS), 1% penicillin/streptomycin, and 1% L-glutamine (all from PAN Biotech, Aidenbach, Germany), as described in [22]. For testing, cells were seeded into 96-well plates (3 × 10^5^ cells/well) and kept at 37 °C and 5% CO_2_. Each well contained 200 µL F-12K cell culture medium in which the concentration of FCS was reduced to 5%. After 24 h, the medium was replaced by the test material preparations [22].

To determine H_2_O_2_ synthesis and release, the test materials were suspended in KRPG buffer (129 mM NaCl, 4.86 mM KCl, 1.22 mM CaCl_2_, 15.8 mM NaH_2_PO_4_, 5–10 mM glucose; pH 7.3–7.4). To determine extracellular release of LDH, GLU, and TNFα, the test materials were suspended in serum-free F-12K cell culture medium (in F-12K assay medium). The suspensions were diluted in the respective fluids to achieve a concentration of 180 µg/mL KRPG buffer or F-12K assay medium, ultrasonicated at 50 W for 10 s with a probe (VibraCell^TM^, Sonics & Materials, Danbury, CT, USA), and serially diluted with KRPG buffer or F-12K assay medium to achieve concentrations of 45, 22.5, 11.2, and 5.6 µg/mL. All tests were performed in triplicate.

#### 4.3.1. H_2_O_2_ Synthesis

H_2_O_2_ synthesized by NR8383 AMs and released into the supernatant was quantified in the Amplex Red^®^ assay as described by Wiemann et al. [22]; zymosan (180 µg/mL) was used as a positive control (all reagents: Sigma-Aldrich). After 90 min incubation, formation of resorufin was determined. Optical density was measured photometrically (Tecan Infinite F200Pro; absorption: 570 nm; reference value: 620 nm), corrected for background absorbance of cell free-particle controls and converted into absolute concentrations of H_2_O_2_ using the molar extinction coefficient of resorufin (54,000 L mol^−1^ cm^−1^).

#### 4.3.2. LDH and GLU Release

LDH activity was determined after 16 h test material incubation using a Roche Cytotoxicity Kit (Sigma-Aldrich) as described by the manufacturer. The induced color change was measured photometrically with an Infinite F200Pro plate reader (Tecan Deutschland GmbH, Crailsheim, Germany). To measure GLU activity, 50 µL of the supernatant (sampled after 16 h incubation) was incubated under standard cell culture conditions with 100 µL 0.2 M sodium acetate buffer (pH 5) containing 13.3 mM p-nitrophenyl-d-glucuronide (Sigma-Aldrich) and 0.1% Triton X-100. The reaction was terminated after 2 h by addition of 100 µL 0.2 M sodium hydroxide. Optical density was measured with a plate reader at 405 nm. Both LDH- and GLU-based values were corrected for cell-free adsorption and normalized to the results of the positive control (0.1% Triton X-100 in F-12K assay medium).

#### 4.3.3. TNFα Release

The amount of released TNFα was determined using the rat TNF-alpha Quantikine ELISA Kit (Bio-Techne GmbH, Wiesbaden-Nordenstadt, Germany) as specified by the manufacturer. F-12K assay medium served as vehicle control. As positive control, the TNFα-forming capacity of NR8383 cells was confirmed by stimulation with lipopolysaccharide (0.1 µg/mL; Sigma-Aldrich).

#### 4.3.4. Overall Evaluation of In Vitro Test Results to Distinguish between Passive and Active Test Materials

The lowest concentration at which a significant effect (*cf.* Section 4.5) was recorded for a given parameter was termed in vitro LOAEC, and it was expressed in both nominal particle mass–based metrics (µg/mL) and nominal and calculated effective concentration particle surface area–based metrics (mm^2^/mL).

A test material concentration threshold of <6000 mm^2^/mL was applied to rule out effects that were caused only under cellular overload conditions [22], i.e., to distinguish passive (MG3) from active (MG4) test materials according to the DF4nanoGrouping [32,33]. This threshold was estimated as corresponding to the highest particle surface area–based concentration that does not yet result in particle overload of the cultured NR8383 AMs, and was further calculated to correspond to the in vivo threshold value for lung cell overload conditions in the rat STIS [22].

To assign the test materials as either passive (MG3) or active (MG4), the in vitro LOAECs obtained for the four parameters (i.e., release of H_2_O_2_, GLU, LDH, or TNFα) were compared to the threshold value of 6000 mm^2^/mL. Any significant LOAEC recorded *below* this threshold value was interpreted as a biologically relevant, test material–specific cellular effect. Significant LOAECs that were recorded only at concentrations exceeding the threshold were interpreted as not test material–specific, but as being caused by the cellular overload. Test materials were assessed as active (MG4) if at least two of the four parameters yielded significant results under non–cellular-overload conditions (i.e., at concentrations below the threshold); they were assessed as passive (MG3) if they yielded 0 or 1 significant result at concentrations below this threshold [22].

### 4.4. In Vivo Rat Intratracheal Instillation Study

The in vivo rat intratracheal instillation study was performed in an Association for Assessment and Accreditation of Laboratory Animal Care–approved laboratory of the Experimental Toxicology and Ecology of BASF SE, Ludwigshafen, Germany, in accordance with the German Animal Welfare Act and the effective European Union Directive. Details on the in vivo rat intratracheal instillation study have been published by Maser et al. [59], who used lung and bone marrow cells from these animals for genotoxicity testing.

Bolus doses of 360 µg 55 nm and 15 nm SiO_2_ in 500 µL 0.9% NaCl (i.e., 720 µg/mL) were administered to Wistar rats (Crl:WI; Charles River, Germany), 8 weeks old on the day of instillation, by single intratracheal instillation. For this purpose, 8 animals per test group and 8 control animals were deeply anesthetized by isoflurane and fixed in an appropriate application holder. Control animals were treated with 0.9% NaCl. Correct insertion of the cannula into the trachea was controlled by visual inspection to avoid insertion into the pharynx. Clinical observation of the animals was performed before and after the instillation procedure and once daily thereafter. The body weight of the animals was assessed before instillation, on the next day, and before gross necropsy. Three days after instillation, the animals were euthanized with Narcoren^®^ (100 mg/kg body weight pentobarbital; Boehringer, Ingelheim, Germany), and 5 animals per test group were used to determine blood and BALF parameters, whereas 3 animals per test group were assigned for pathological and histopathological examination. All tissues with gross lesions and all organs associated with the respiratory tract (nasal cavity, larynx, trachea with bifurcation, lungs, and mediastinal lymph nodes) were embedded in paraffin, sectioned, and stained with hematoxylin-eosin for histopathological evaluation by light microscopy.

### 4.5. Statistical Analyis

The significance of the in vitro data (NR8383 AM assay) was assessed for all 4 cellular parameters (release of H_2_O_2_, LDH, GLU, or TNFα). Values were compared to the vehicle control data using Bonferroni multiple testing and Bonferroni correction. To analyze values from the in vivo rat intratracheal instillation study (clinical pathology data), 2-sided Mann-Whitney U test was used; *p*-values ≤ 0.05 were considered significant.

## 5. Conclusions

Four colloidal amorphous SiO_2_ that differed only by PPS (9 nm SiO_2_, 15 nm SiO_2_, 30 nm SiO_2_, and 55 nm SiO_2_) elicited concentration-dependent release of LDH, GLU, and TNFα (and H_2_O_2_) in the in vitro NR8383 AM assay. Further, particle size and, hence, surface area dependency of the cellular effects of these nanomaterials was clearly recognizable. The NR8383 AM assay, the recommended in vitro test method to determine a nanomaterial’s ability to induce cellular effects in Tier 2 of the DF4nanoGrouping [32,33], proved useful to assign the differently sized colloidal SiO_2_ as active (MG4) nanomaterials. Applying the two-out-of-four prediction model to evaluate the effective concentration-based test results, all four colloidal SiO_2_ were assigned as active (MG4). This assignment was also indicated by their dispersibility, a DF4nanoGrouping Tier 2 grouping criterion [32,33]. All four test materials remained well dispersed in protein-free media (with average agglomeration numbers below the DF4nanoGrouping threshold of 3 indicating activity (MG4)).

The in vitro test results are consistent with the findings from the intratracheal instillation study, where both 15 nm and 55 nm SiO_2_ elicited pulmonary effects after administration of single bolus doses reflecting the 15 nm SiO_2_ lung burden immediately after 5-day aerosol exposure in the rat STIS, as well as with available rat STIS data for 15 nm SiO_2_ [15]. The particle size dependency of in vitro effects was confirmed in the intratracheal instillation study, where the smaller 15 nm SiO_2_ also induced more pronounced effects than the larger 55 nm SiO_2_.

Previous work [22] highlighted the usefulness of the NR8383 AM assay in distinguishing passive (MG3) from active (MG4) nanomaterials. Therefore, this assay may become relevant for hazard and risk assessment of nanomaterials (Box 3). The present study further confirms the usefulness of the NR8383 AM assay to assess colloidal SiO_2_ while underlining the need to determine the effective concentration of such nanomaterials with limited gravitational settling. However, the NR8383 AM assay still has some uncertainties if the test materials do not readily come into contact with the cultured cells, as is the case for well-dispersed colloidal SiO_2_. For these test materials that poorly sedimented within the 16 h incubation period, calculating the effective concentration proved necessary for evaluation of the in vitro test results in spite of some uncertainties underlying these calculations. In the present study, the ISDD model [66] was applied to calculate the effective concentration. While this model assumes a probability of 1 that particles that come close to the cells adhere to them [66], the probability might be much lower for strongly negatively charged colloidal amorphous SiO_2_. Further research is merited to develop methodologies to quantitatively measure effective concentrations, taking into account sedimentation and diffusion of particles and their subsequent adhesion to the bottom of the culture wells or cultured cells.

Box 3 **Further hazard and risk assessment of nanomaterials assigned to one of the main groups (MG1–MG4) of the DF4nanoGrouping [32,33]).**

MG1—soluble nanomaterials: The further hazard and risk assessment can rely on read-across to the dissolved materials [32,33].

MG2—biopersistent high aspect ratio nanomaterials: The further hazard and risk assessment should address their potential fibre toxicity [101,102,103].

MG3—passive nanomaterials: For risk assessment, the general threshold limit value for dust is sufficient to ensure occupational safety upon long-term exposure since these nanomaterials do not elicit material-specific effects, but only cause effects under pulmonary overload conditions [104].

MG4—active nanomaterials: Merit in-depth investigations. For risk assessment, occupational exposure limits (lower than for dusts) should be established on a case-by-case basis [7].

## Figures and Tables

**Table 1:** Characterization of colloidal amorphous SiO_2_ nanomaterials (adapted from Maser et al. [59] and Arts et al. [33]).

Parameter (Test Method)	Unit	9 nm SiO_2_	15 nm SiO_2_^g^	30 nm SiO_2_	55 nm SiO_2_
Specific surface area (specified by producer)	m^2^/g	300	200	100	50
Solid concentration	%	30	40	45	50
Purity/crystalline phase (XRD) ^a^	%/qualitative	not determined	>99%/amorphous	not determined	>99%/amorphous
Appearance	qualitative	opalescent	opalescent	milky	milky
pH value (specified by producer)	–	10	10	10	10
Primary particle size (specified by producer)	nm	9	15	30	55
Particle size ± SD in water (intensity-averaged, DLS) ^b^	nm	26 ± 0.7	48 ± 0.4	85 ± 0.6	117 ± 0.4
Particle size in KRPG medium (AUC)	nm	13	24	41	82
Particle size in F-12K medium (AUC) ^c^	nm	13	21	47	87
Particle size in 0.9% NaCl (AUC) ^c^	nm	not determined	26	not determined	77
Particle size in DMEM + 10% FCS (AUC)	D_50_ (nm)	not determined	289	not determined	384
Water solubility (pH 7, static, filtration 5 kDa) ^d^	mg/L	48	43	19	7
Dissolution rate in lysosomal medium (pH 4.5 PSF, flow-through 5 kDa)	ng/cm²/h	0.034	0.044	0.066	0.088
Zeta potential ± SD (Zetasizer)	mV	−43	−48	−55	−50
Surface chemistry (XPS) ^a^	atomic %	O 69 | Si 28 | C 2 | Na 1	O 65 | Si 29 | C 4 | Na 1 | N 0.6	O 68 | Si 29 | C 2 | Na 1	O 53 | Si 40 | C 5 | Na 2 | N 0.3 | Cl 0.5
Surface organic contaminations (SIMS) ^a^	qualitative	not determined	SiO_2_ cluster; organics, traces of surfactant	not determined	SiO_2_ cluster; less organics than 15 nm SiO_2_
Specific surface reactivity ± SD (sBOD at 1 m²/mL)	nM TEU/m² nanomaterial	13.9 ± 0.3 nonoxidative	14 ± 0.5 nonoxidative	13.2 ± 0.0 nonoxidative	7.5 ± 0.2 nonoxidative
Surface reactivity (ESR + CPH) ^e,f^	relative to D_2_O	not determined	4/p-f s: 0.88	not determined	not determined
Surface ROS generation (ESR + DMPO) ^e,f^	relative to D_2_O	not determined	11/p-f s: 6.3	not determined	not determined

Abbreviations: AAN: average agglomeration number; AUC: analytical ultracentrifugation; DLS: dynamic light scattering; DMEM: Dulbecco’s Modified Eagle Medium; ESR + CPH: electron spin resonance making use of centrophenoxine spin traps; ESR + DMPO: electron spin resonance making use of dimethy–pyrroline–N-oxide spin traps; FCS: fetal calf serum; KRPG: Krebs–Ringer phosphate glucose; p-f s: particle-free supernatant; PSF: phagolysosomal simulant fluid; ROS: reactive oxygen species; sBOD: surface-induced biological oxidative damage; SD: standard deviation; SIMS: secondary ion mass spectrometry; TEU: Trulox equivalent unit; XPS: x-ray photoelectron spectroscopy; XRD: x-ray diffusion. ^a^: Published by Schaefer et al. [63]. ^b^: The DLS-based particle size in water is consistent with earlier data on a subset of the test materials (Schaefer et al. [63]). The DLS data recorded here were newly generated to ensure comparability of the data. ^c^: For all test materials, a polydispersity index (D_90_ − D_10_)/D_50_ around 1 was calculated, indicating a broad particle size distribution. ^d^: Hypothetically, the soluble content might be a mix of ions, cluster, and smallest particles. Since the 5kDa filter cutoff corresponds to pore sizes below 1 nm, all compounds able to pass the filter are not nanomaterials. ^e^: Published by Izak-Nau and Voetz [64]. ^f^: Surface reactivity and ROS formation were determined relative to the reference material deuterium oxide (D_2_O; ^2^H_2_O). Assuming 30% variability of the methodology, only measurements >1.3 are considered relevant, taking into account that this value serves as a guiding principle, not an absolute value. ^g^: Applying Langmuir isotherm approximation, Chen et al. [65] assessed the following infinite dilution adsorption descriptors for 15 nm SiO_2_: r: 0.61; p: −0.32; a: 1.16; b: −1.79; v: 1.15. The parameters r, p, a, b, and v represent the five major molecular interaction forces in the nanoparticle adsorption processes, i.e., the five descriptors of the biological surface adsorption index: lon-pair electrons, polarity/polarizability, hydrogen-bond donor, hydrogen-bond acceptor, and London dispersion, respectively.

**Table 2:** Particle mass–based test material concentrations juxtaposed with corresponding nominal and effective surface area–based concentrations of the test materials.

Test Material	Surface Area (BET Method)	Effective Concentration (ISDD Model) ^b^	Surface Area-Based Test Material Concentrations ^a^							
			5.6 µg/mL Corresponds to:		11.2 µg/mL Corresponds to:		22.5 µg/mL Corresponds to:		45.0 µg/mL Corresponds to:	
			Nominal Concentration	*Effective Concentration*	Nominal Concentration	*Effective Concentration*	Nominal Concentration	*Effective Concentration*	Nominal Concentration	*Effective Concentration*
	m^2^/g	%	mm^2^/mL	mm^2^/mL	mm^2^/mL	mm^2^/mL	mm^2^/mL	mm^2^/mL	mm^2^/mL	mm^2^/mL
**55 nm SiO_2_**	**50**	15.8	280	44	560	88	1125	178	2250	356
**30 nm SiO_2_**	**100**	17.4	560	97	1120	195	2250	392	4500	783
**15 nm SiO_2_**	**200**	24.6	1120	276	2240	551	4500	1107	9000	2214
**9 nm SiO_2_**	**300**	31.0	1680	521	3360	1042	6750	2093	13,500	4185

Abbreviations: BET: Brunauer, Teller, and Emmett; ISDD: in vitro sedimentation, diffusion, and dosimetry. ^a^: To convert the nominal particle mass–based test material concentrations (µg/mL) into particle surface area–based concentrations (mm^2^/mL), they were multiplied with the respective test material’s surface area (m^2^/g) as assessed by the BET method [22]. ^b^: The effective concentration of the test materials in F-12K medium after 16 h incubation was determined using the ISDD model [66]. For the modelling, the following parameters were set: dish depth: 0.006 m; volume: 0.2 mL; viscosity (H_2_O, 37 °C): 0.00074 mPa x s; temperature: 310 K; SiO_2_ density: 2.3 g/mL; particle size in F-12K medium (*cf.* Table 1) equals agglomerate size; incubation time: 16 h; packing factor: 0.64; fractal dimension: 42,796.00.

**Table 3:** Effects elicited by 55 nm SiO_2_, 30 nm SiO_2_, 15 nm SiO_2_, and 9 nm SiO_2_ in the NR8383 AM assay: overall evaluation.

Test Material	Surface Area (m^2^/g; BET Method)	In Vitro LOAEC (µg/mL) ^a^				In Vitro LOAEC (mm^2^/mL) ^a^								Number of Positive Parameters	
		H_2_O_2_	LDH	GLU	TNFα	H_2_O_2_		LDH		GLU		TNFα			
		Nominal Concentration				Nom. Conc.	Eff. Conc.	Nom. Conc.	Eff. Conc.	Nom. Conc.	Eff. Conc.	Nom. Conc.	Eff. Conc.	Nom. Conc.	Eff. Conc.
**55 nm SiO_2_**	**50**	n.s.	45	45	n.s.	n.s.	n.s.	2250	356	2250	356	n.s.	n.s.	2	2
**30 nm SiO_2_**	**100**	n.s.	45	45	45	n.s.	n.s.	4500	783	4500	783	4500	783	3	3
**15 nm SiO_2_**	**200**	45	22.5	45	45	9000	2214	4500	1107	9000	2214	9000	2214	1	4
**9 nm SiO_2_**	**300**	45	22.5	22.5	22.5	13,500	4185	6750	2093	6750	2093	6750	2093	0	4
**15 nm SiO_2_^b^**	**200**	45	22.5	45	22.5	9000		4500		9000		4500		2	

Abbreviations: BET: Brunauer, Emmett, and Teller; GLU: β-glucuronidase; Eff. Conc.: effective concentration; ISDD: in vitro sedimentation, diffusion, and dosimetry; LDH: lactate dehydrogenase; LOAEC: lowest observed adverse effect concentration; MG: main group; n.s.: not significant; Nom. Conc.: nominal concentration; TNFα: tumor necrosis factor alpha. ^a^: For all four parameters (H_2_O_2_, LDH, GLU, and TNFα release), the in vitro LOAECs (i.e., the lowest concentrations that elicited significant effects; *cf.* Table S2) are shown as nominal concentrations (expressed as mass/volume (μg/mL) and surface area/volume (mm^2^/mL)) and as effective concentrations (expressed as surface area/volume (mm^2^/mL)) (*cf.* Table 2). Surface area/volume-based in vitro LOAECs that undercut the threshold of 6000 mm^2^/ mL were assessed as positive (highlighted with gray shading). The number of positive parameters was added to assign test materials as either active (MG4) or passive (MG3) in accordance with the DF4nanoGrouping [22,32,33]. Test materials were assigned as active (MG4) if at least two of the four parameters were positive (gray shading in the far-right column). ^b^: Published by Wiemann et al. [22].

**Table 4:** Effects of 55 nm and 15 nm SiO_2_ in a rat intratracheal instillation study: terminal body weight and organ weight (three animals per group).

		Control Group	55 nm SiO_2_		15 nm SiO_2_	
			(g)	% dev	(g)	% dev
Body weight	Mean	213.47	223.13	5	216.2	1
	SD	11.73	7.5		5.21	
Kidney	Mean	1.58	1.7	8	1.6	1
	SD	0.16	0.1		0.11	
Liver	Mean	6.54	6.95	6	7.07	8
	SD	0.69	0.27		0.62	
Lung	Mean	0.85	0.93	10	1.17	38
	SD	0.04	0.09		0.11	
Spleen	Mean	0.51	0.54	7	0.61	19
	SD	0.08	0.04		0.06	

Abbreviations: dev: deviation (from control group); SD: standard deviation.

**Table 5:** In vivo rat intratracheal instillation study assessing 55 nm and 15 nm SiO_2_: hematology and bronchoalveolar lavage fluid parameters (five animals per group).

Parameter	55 nm SiO_2_		15 nm SiO_2_	
**Hematology**				
WBC	0.9 ± 0.4		1.1 ± 0.1	
RBC	1.0 ± 0.0		1.0 ± 0.0	
HGB	1.0 ± 0.0		1.0 ± 0.0	
HCT	1.0 ± 0.0		1.0 ± 0.0	
MCV	1.0 ± 0.0		1.0 ± 0.0	
MCH	1.0 ± 0.0		1.0 ± 0.0	
MCHC	1.0 ± 0.0		1.0 ± 0.0	
PLT	0.9 ± 0.0	**	1.0 ± 0.0	
NEUT	1.0 ± 0.4		1.9 ± 0.4	*
LY	0.9 ± 0.4		1.0 ± 0.1	
MONO	0.9 ± 0.4		1.1 ± 0.1	
EO	1.0 ± 0.3		1.2 ± 0.5	
BASO	1.0 ± 1.0		1.0 ± 1.0	
LUC	1.0 ± 0.5		1.0 ± 0.3	
**Bronchoalveolar lavage fluid**				
Total cells	2.8 ± 0.5	**	5.5 ± 1.0	**
MPH	1.1 ± 0.4		1.3 ± 0.5	
LY	155.7 ± 36.3	**	391.7 ± 197.7	**
PMN	66.1 ± 29.0	**	151.1 ± 27.8	**
MONO	+		+	*
EO	9.8 ± 9.8	*	92.7 ± 75.9	**
ATY	+	*	+	**
Total protein	1.9 ± 0.5	*	4.7 ± 0.4	**
GGT	1.5 ± 0.6		4.2 ± 1.1	**
LDH	2.7 ± 1.0	**	5.0 ± 1.6	**
ALP	1.8 ± 0.5	**	2.9 ± 0.7	**
NAG	1.0 ± 0.3		2.0 ± 0.4	**

All values are expressed as fold changes compared to concurrent controls (mean ± standard deviation). Statistical analysis was performed by two-sided Mann-Whitney U test (* *p*-value ≤ 0.05; ** *p*-value ≤ 0.01). Abbreviations: ALP: alkaline phosphatase; ATY: atypical cell; BASO: basophil; EO: eosinophil; GGT: γ-glutamyltransferase; HCT: packed cell volume; HGB: hemoglobin; LDH: lactate dehydrogenase; LUC: large unstained cell; LY: lymphocyte; MCH(C): mean corpuscular hemoglobin (concentration); MCV: mean corpuscular volume; MONO: monocyte; MPH: macrophage; NAG: *N*-acetyl-β-glucosaminidase; NEUT: neutrophil; PLT: platelet; PMN: polymorphonuclear neutrophil; RBC: red blood cell; TP: total protein; WBC: white blood cell.

## Supplementary Materials

The following are available online at http://www.mdpi.com/2079-4991/8/3/160/s1: Figure S1: Sedimentation of colloidal amorphous SiO_2_ in Krebs-Ringer phosphate glucose (KRPG) buffer determined by analytical ultracentrifugation. Yellow: 9 nm SiO_2_; gray: 15 nm SiO_2_; orange: 30 nm SiO_2_; blue: 55 nm SiO_2_. Figure S2A: Fraction of deposited dose calculated with the DG model [1] applying the “sticky bottom” assumption. Yellow: 55 nm SiO_2_; gray: 30 nm SiO_2_; orange: 15 nm SiO_2_; blue: 9 nm SiO_2_. Figure S2B: Fraction of deposited dose calculated with the DG model [1] applying the “reflective bottom” assumption. Yellow: 55 nm SiO_2_; gray: 30 nm SiO_2_; orange: 15 nm SiO_2_; blue: 9 nm SiO_2_. Table S1: Analytical ultracentrifugation (AUC) measurements of the proportion of applied test materials that reached the bottom of the vials by sedimentation. Table S2: In vitro NR8383 rat alveolar macrophage assay: Cellular endpoint-specific test results obtained for 55 nm SiO_2_, 30 nm SiO_2_, 15 nm SiO_2_, and 9 nm SiO_2_ (expressed as x-fold changes compared to corresponding medium control).
