# Supplementary material for: In Vitro and In Vivo Short-Term Pulmonary Toxicity of Differently Sized Colloidal Amorphous SiO2
Source: Nanomaterials (Basel). 2018 Mar 13;8(3):160. doi: 10.3390/nano8030160 (PMC5869651; doi:10.3390/nano8030160)
Supplement: Supplementary file 1 [file nanomaterials-08-00160-s001.docx]

**Supplementary Information**

***In vitro* and *in vivo* short-term pulmonary toxicity of differently sized colloidal amorphous SiO_2_**

Martin Wiemann^1#^*; Ursula G. Sauer^2#^; Antje Vennemann^1^; Sandra Bäcker^3^; Johannes-Georg Keller^4^; Lan Ma-Hock^5^; Wendel Wohlleben^4^, and Robert Landsiedel^5^

^1^ IBR R&D gGmbH Institute for Lung Health, Germany; [martin.wiemann@ibe-ms.de](mailto:martin.wiemann@ibe-ms.de)

^2^ Scientific Consultancy – Animal Welfare, Germany; [ursula.sauer@sauerug.de](mailto:ursula.sauer@sauerug.de)

^3^ BASF SE, Human Biomonitoring and Industrial Hygiene, Germany; [sandra.baecker@basf.com](mailto:sandra.baecker@basf.com)

^4^ BASF SE, Advanced Materials Research*,* Germany; [wendel.wohlleben@basf.com](mailto:wendel.wohlleben@basf.com)

^5^ BASF SE, Experimental Toxicology and Ecology, Germany; [lan.ma-hock@basf.com](mailto:lan.ma-hock@basf.com), [robert.landsiedel@basf.com](mailto:robert.landsiedel@basf.com)

***** Correspondence: IBR R&D gGmbH Institute for Lung Health, Mendelstr. 11, 48149 Münster, Germany; [martin.wiemann@ibe-ms.de](mailto:martin.wiemann@ibe-ms.de)


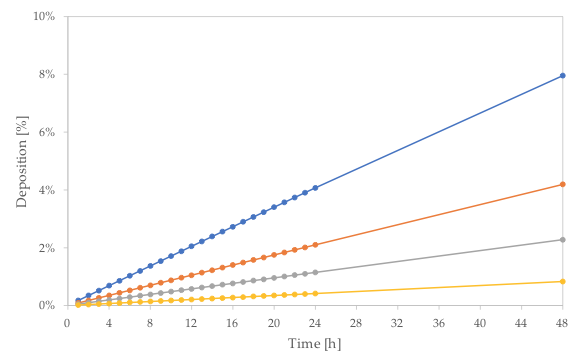


**Figure S1.** Sedimentation of colloidal amorphous SiO_2_ in Krebs-Ringer phosphate glucose (KRPG) buffer determined by analytical ultracentrifugation. Colour legend: Yellow: 9 nm-SiO_2_; grey: 15 nm-SiO_2_; orange: 30 nm-SiO_2_; blue: 55 nm-SiO_2_.


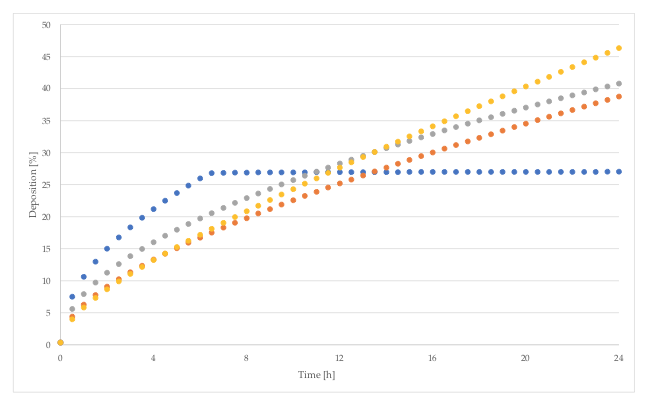


**Figure S2A.** Fraction of deposited dose calculated with the DG-Model [1] applying the ‘sticky bottom assumption’. Colour legend: Yellow: 55 nm-SiO_2_; grey: 30 nm-SiO_2_; orange: 15 nm-SiO_2_; blue: 9 nm-SiO_2_.


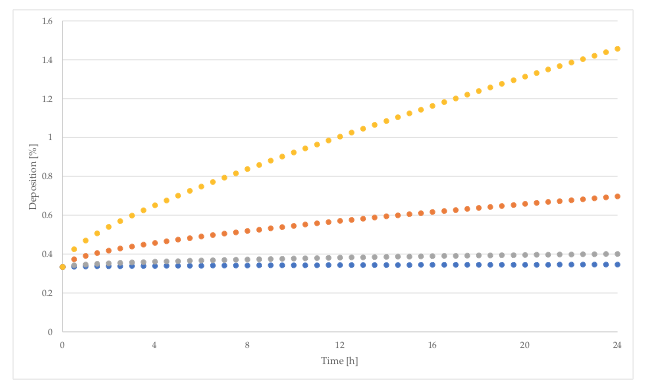


**Figure S2B.** Fraction of deposited dose calculated with the DG-Model [1] applying the ‘reflective bottom assumption’. Colour legend: Yellow: 55 nm-SiO_2_; grey: 30 nm-SiO_2_; orange: 15 nm-SiO_2_; blue: 9 nm-SiO_2_.

1. DeLoid, G.M.; Cohen, J.M.; Pyrgiotakis, G.; Demokritou, P. Preparation, characterization, and in vitro dosimetry of dispersed, engineered nanomaterials. *Nat Protoc* **2017,** *12*, 355-371.

**Table S1.** Analytical ultracentrifugation (AUC) measurements of the proportion of the applied test materials that reached the bottom of the vials by sedimentation.

| **Test material** | **Effective test material dose in KRB medium (AUC) ^a^** | |
| --- | --- | --- |
|  | **Dose that reached cells by sedimentation after 12 h** | **Dose that reached cells by sedimentation after 24 h** |
|  | % | % |
| **55 nm-SiO_2_** | 2.2 | 4.3 |
| **30 nm-SiO_2_** | 1.1 | 2.2 |
| **15 nm-SiO_2_** | 0.6 | 1.2 |
| **9 nm-SiO_2_** | 0.2 | 0.4 |

**Table S2.** *In vitro* NR8383 rat alveolar macrophage assay: Cellular endpoint-specific test results obtained for 55 nm-SiO_2_, 30 nm-SiO_2_, 15 nm-SiO_2_, and 9 nm-SiO_2_ (expressed as x-fold changes as compared to corresponding medium control).


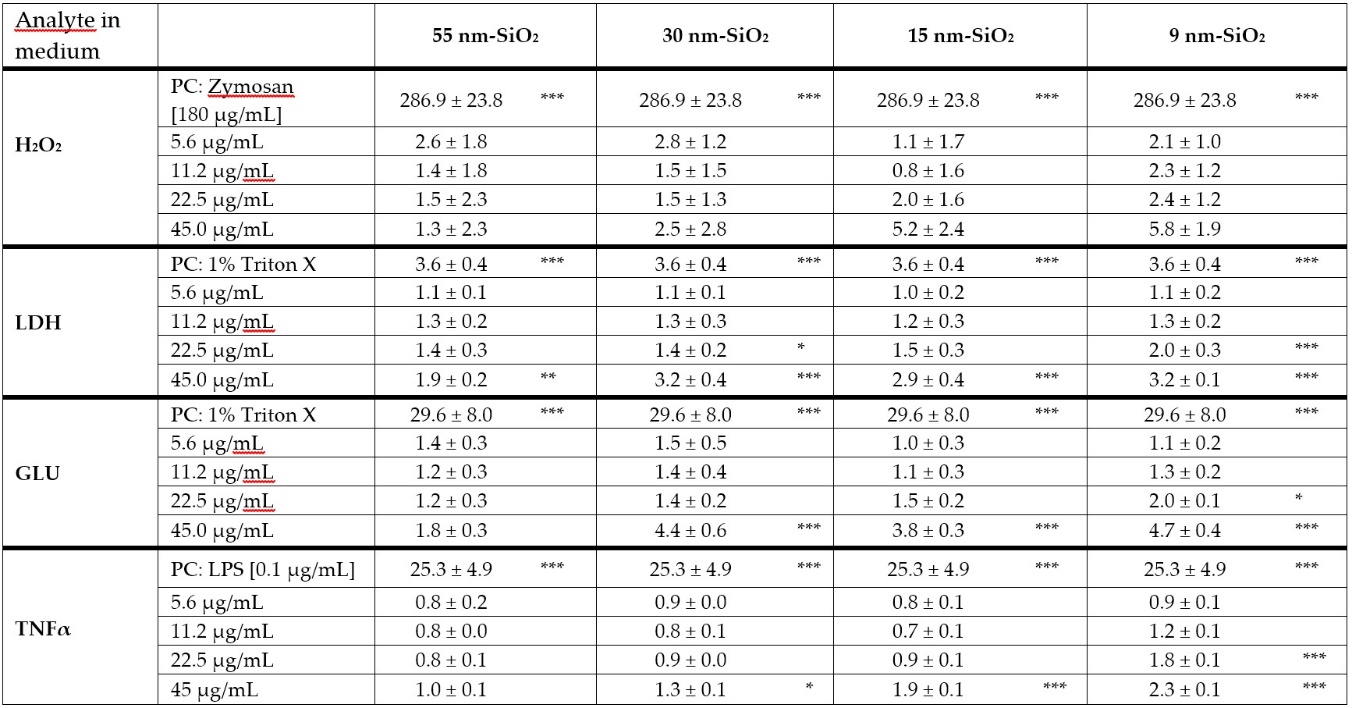
Abbreviations: GLU: β-glucuronidase; LDH: Lactate dehydrogenase; LPS: Lipopolysaccharide; PC: Positive control; TNFα: Tumour necrosis factor alpha. PC: positive control; depending on the assay the PC consisted in either zymosan treatment (H_2_O_2_), lysis with Triton X-100 (LDH, GLU), or stimulation with LPS (TNFα).

Values are expressed as means of three independent test runs ± standard deviation. Bonferroni multiple testing and Bonferroni correction were applied to determine statistical significance of effects (p-value ≤ 0.05: *; p-value ≤ 0.01: **; p-value ≤ 0.001: ***).

**Table S3.** *In vitro* NR8383 rat alveolar macrophage assay: Cellular endpoint-specific test results obtained for 55 nm-SiO_2_, 30 nm-SiO_2_, 15 nm-SiO_2_, and 9 nm-SiO_2_ (expressed in absolute values).


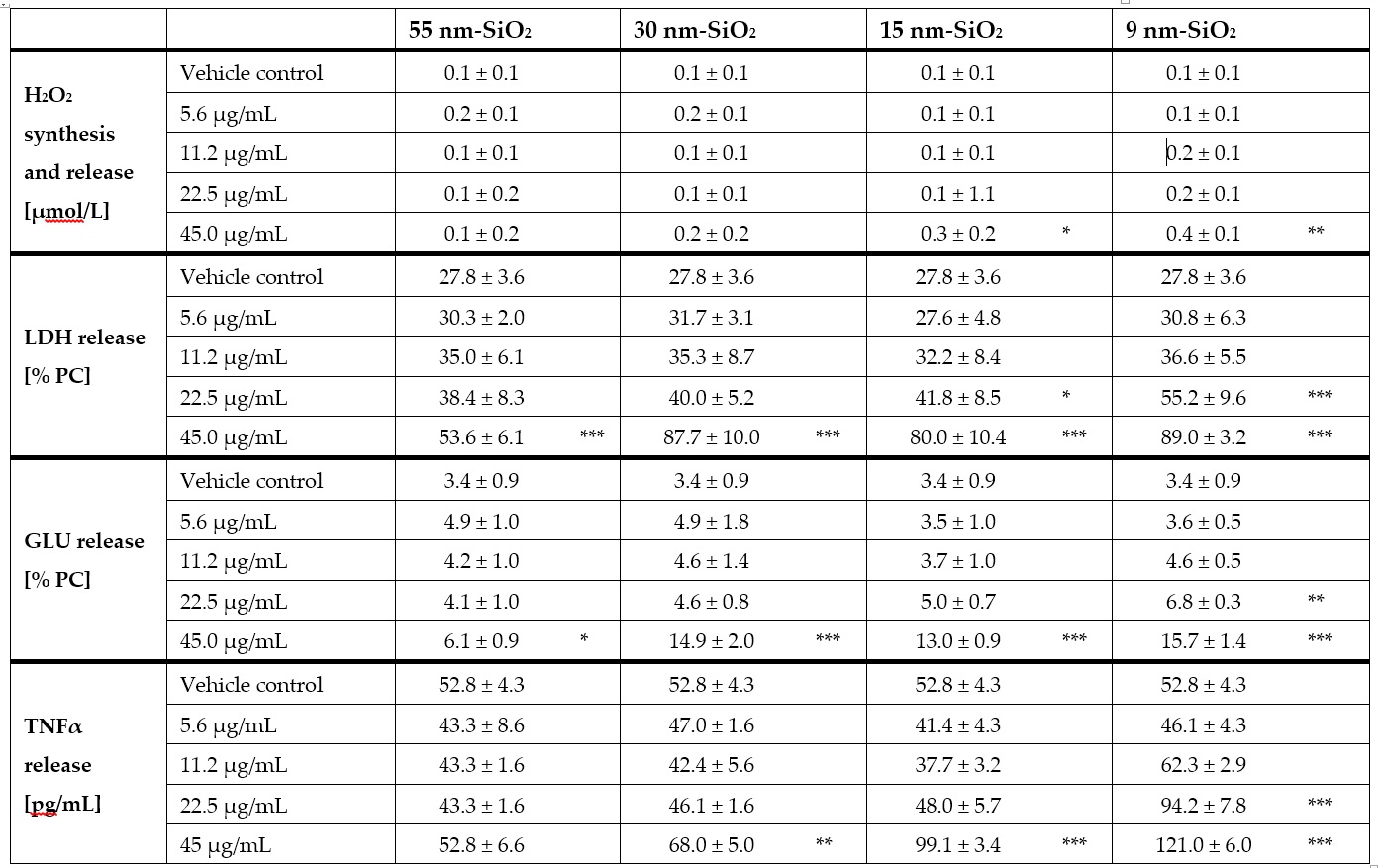


Abbreviations: GLU: β-glucuronidase; LDH: Lactate dehydrogenase; PC: Positive control; TNFα: Tumour necrosis factor alpha. Values are expressed as means of three independent test runs ± standard deviation. Bonferroni multiple testing and Bonferroni correction were applied to determine statistical significance of effects (p-value ≤ 0.05: *; p-value ≤ 0.01: **; p-value ≤ 0.001: ***).
